# Supplementary material for: Exploring Evolutionary Relationships Across the Genome Using Topology Weighting
Source: Genetics. 2017 Mar 23;206(1):429–38. doi: 10.1534/genetics.116.194720 (PMC5419486; doi:10.1534/genetics.116.194720)
Supplement: Supplementary file 2 [file 429FileS2.pdf]

Supplementary Figures

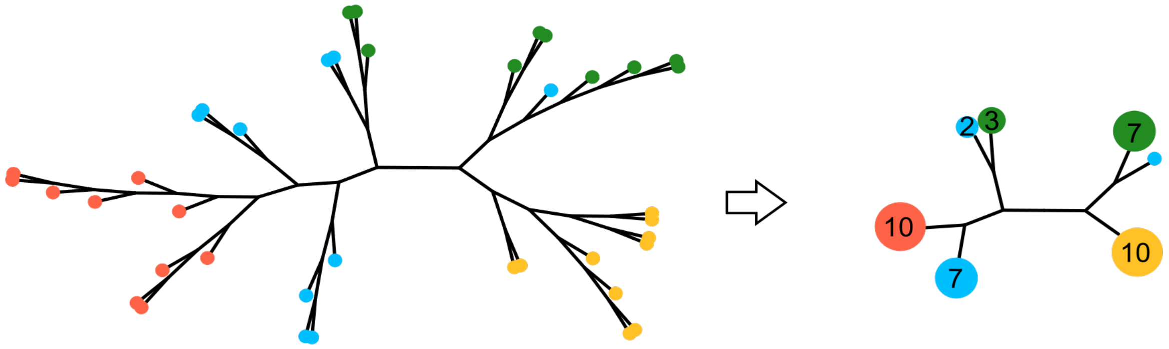

Figure S1. Tree Simplification

The full tree (left) has four defined taxa of ten samples each, indicated by different colours. This equates to 10,000 (10^4) unique sub-trees that include a single individual from each taxon. *Twisst* reduces the number of unique sub-trees to count by collapsing clades and weighting them by the number of individuals of each taxon present (and adjusting branch lengths accordingly). In this example, after this process, there remain only 6 unique sub-trees to count (3x2x1x1).

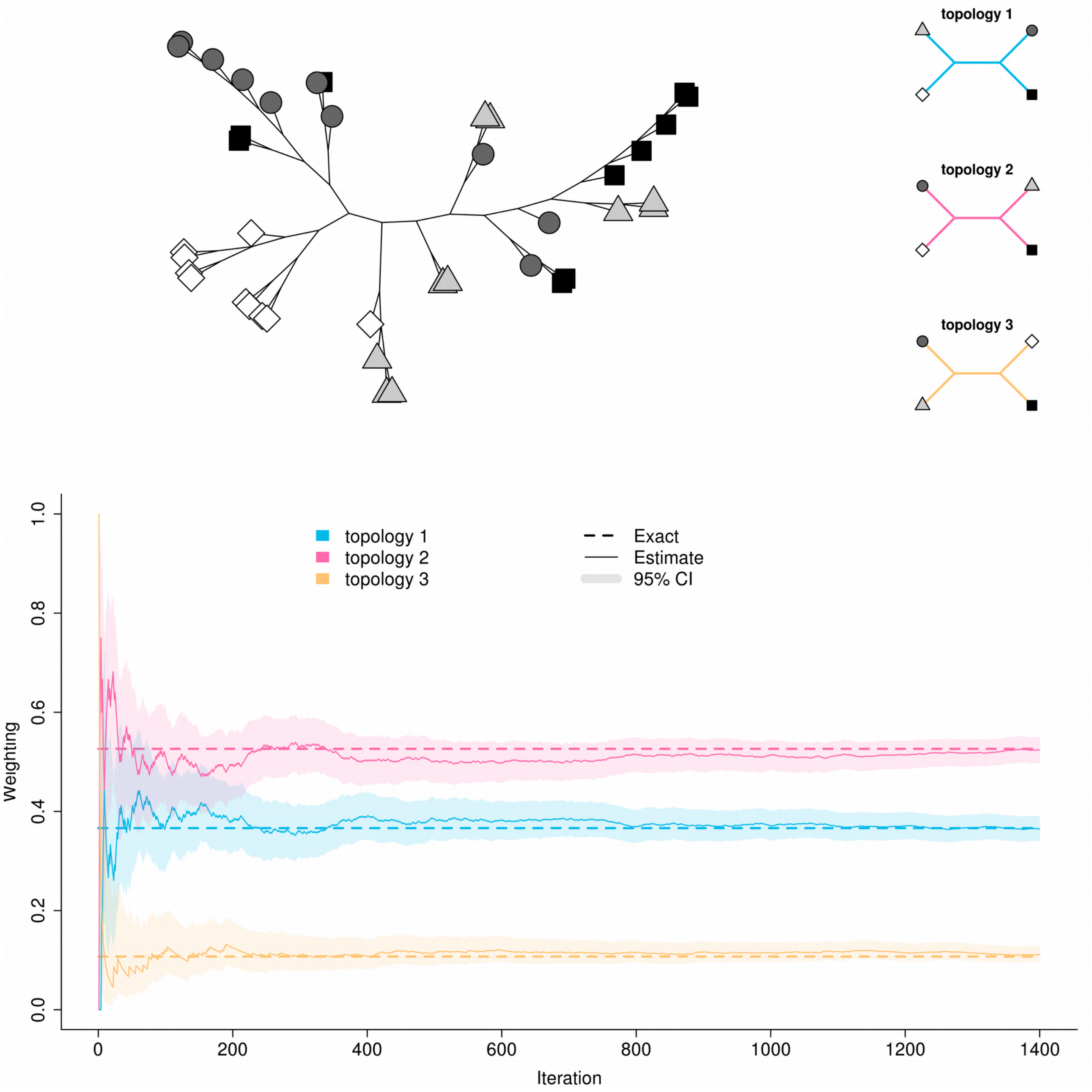

Figure S2. Approximate weighting by random sampling

This example tree has four defined taxa indicated by different symbols. There are three possible taxon topologies (top right). The graph shows the estimated weighting for each topology (Y-axis) after randomly sampling a number of sub-trees from the full tree (X-axis). The 95% binomial confidence interval (calculated using the Wilson method), is shaded. Dashed lines show the true exact weightings computed by sampling all 10,000 sub-trees.

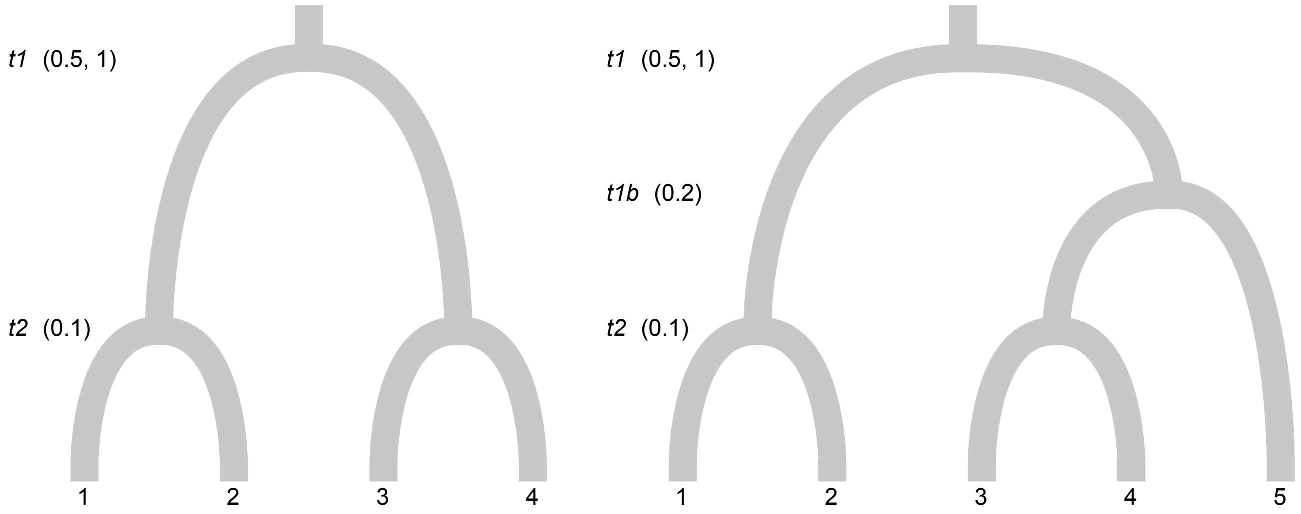

Figure S3. Demographic scenarios for power simulations

Four (left) and five (right) population simulations were performed. Split times are shown, note that two different values were tested for split time *t1* in both scenarios.

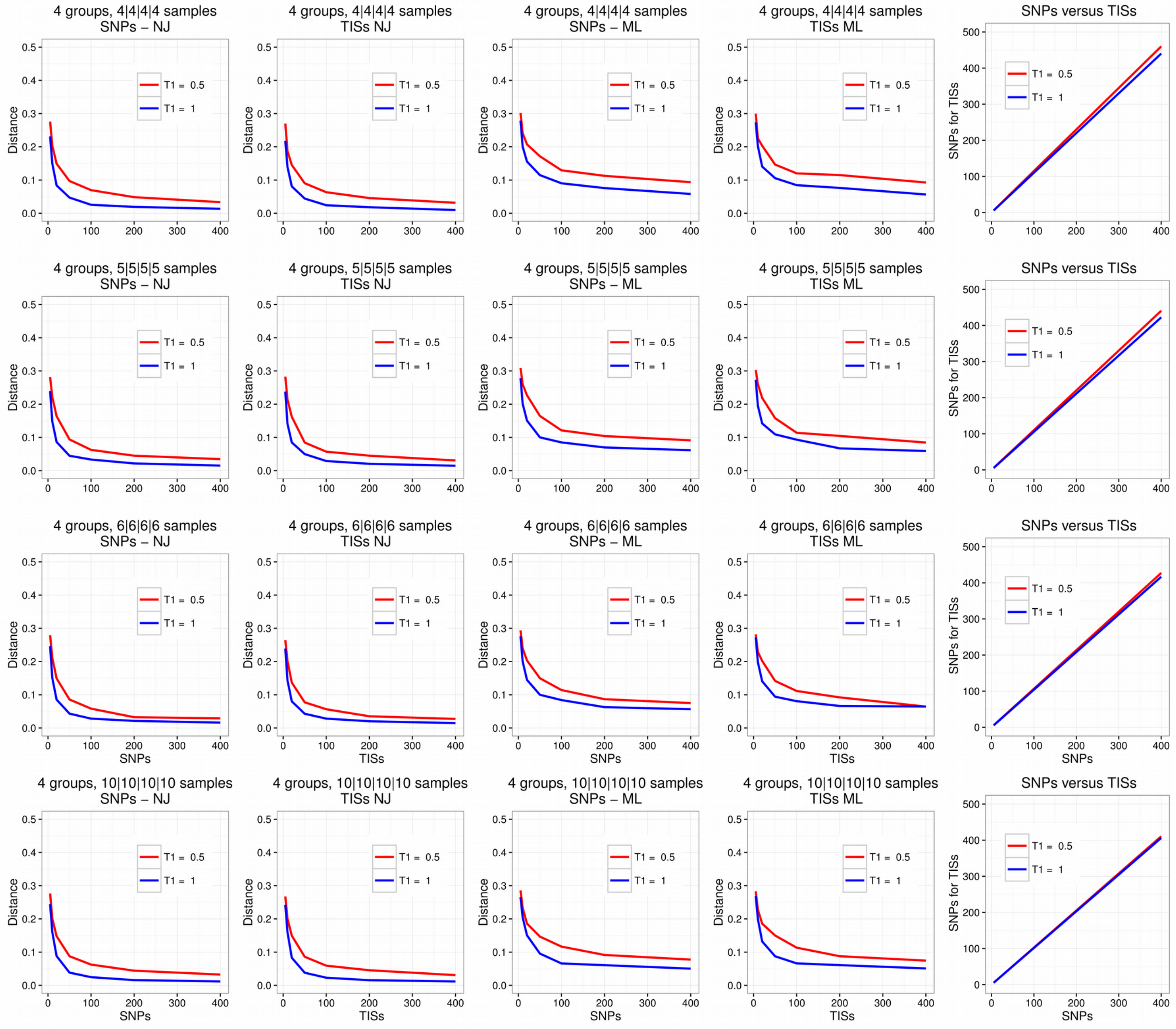

Figure S4. Power analyses with four equal-sized groups

Each plot in the first four columns shows the error rate, calculated as a scaled euclidean distance between the true and inferred weightings (Y-axis), plotted against the number of sites used for tree inference (X-axis). The simulated sequences were truncated to create a sequence of the correct length for tree inference. Truncation was performed either after X SNPs were observed (columns 1 and 3) or after X 'taxon-informative sites' (TISs) had been observed (columns 1 and 4). The final column gives the number of SNPs required (Y-axis) to observe a certain number of TISs (X-axis). The first two columns show results after tree inference using neighbour joining (NJ) and the next two columns show results after tree inference using maximum likelihood (ML). Each row represents a distinct sampling strategy (four groups of four samples, four groups of five samples etc.). Red and blue lines indicate the two different demographic scenarios tested, with a different split time *t1* (see Fig. S3).

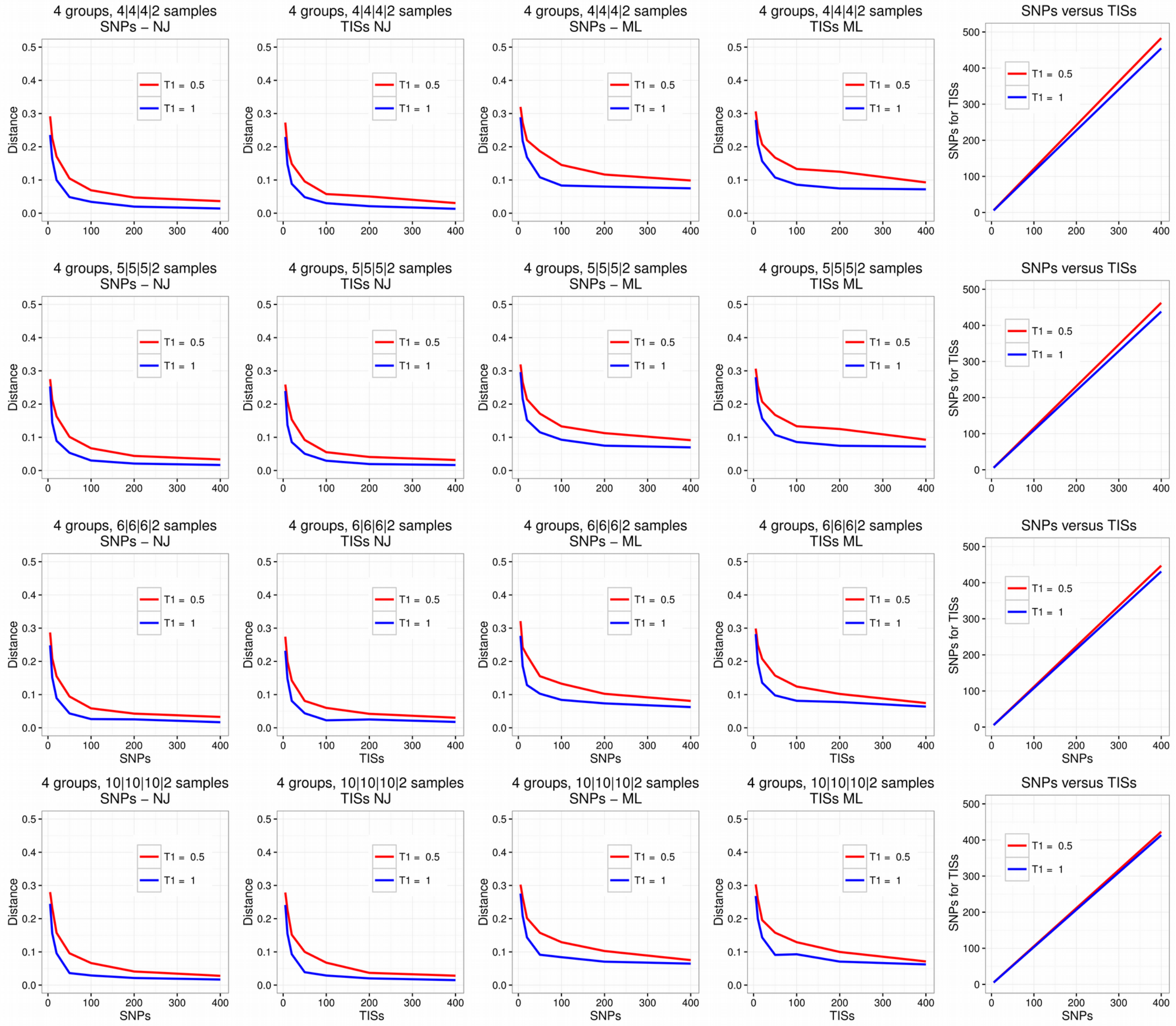

Figure S5. Power analyses with four groups of different sizes

As in Fig. S4, except for groups of different sizes.

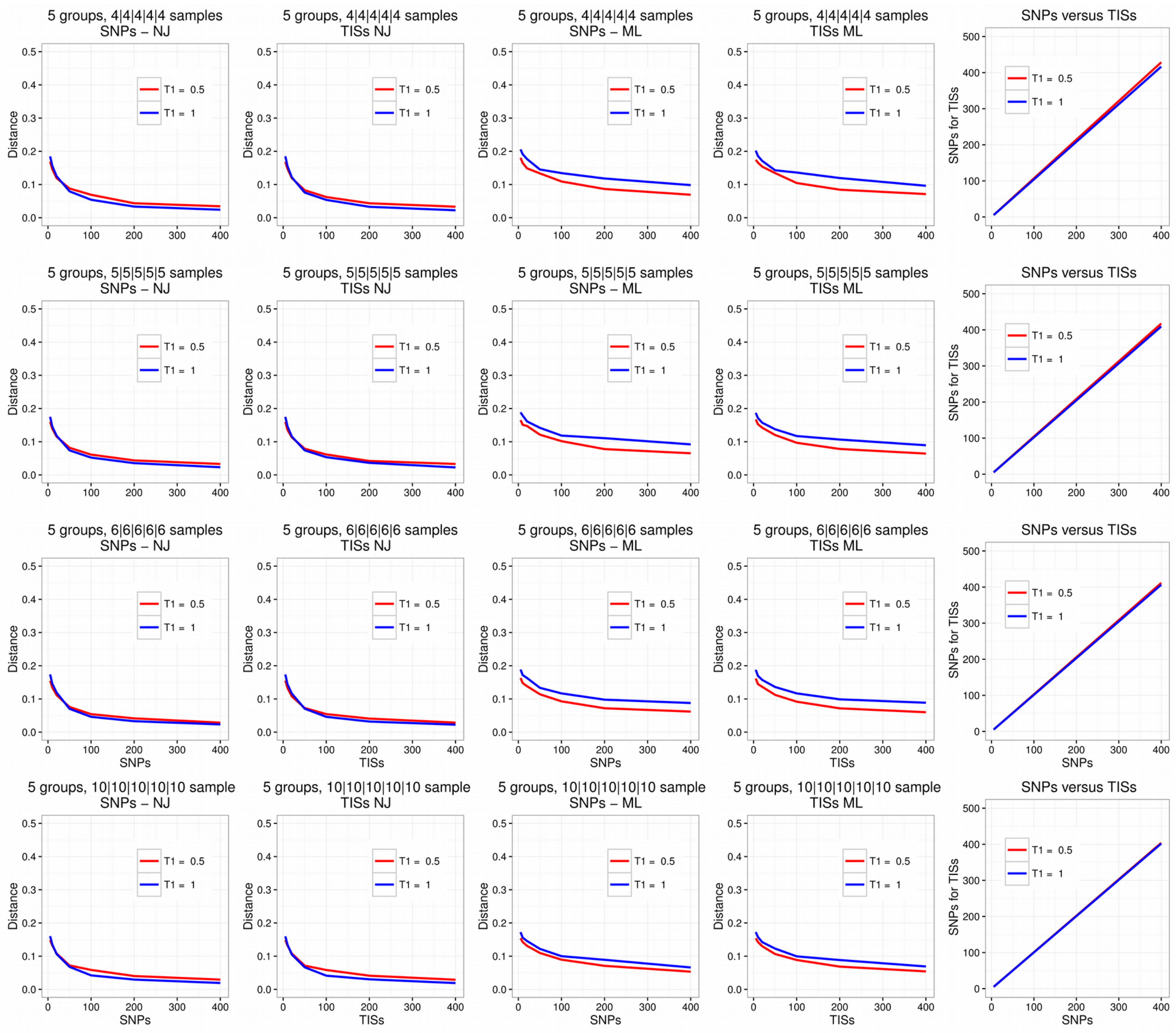

Figure S6. Power analyses with five equal-sized groups

As in Fig. S4, except for simulations with five groups.

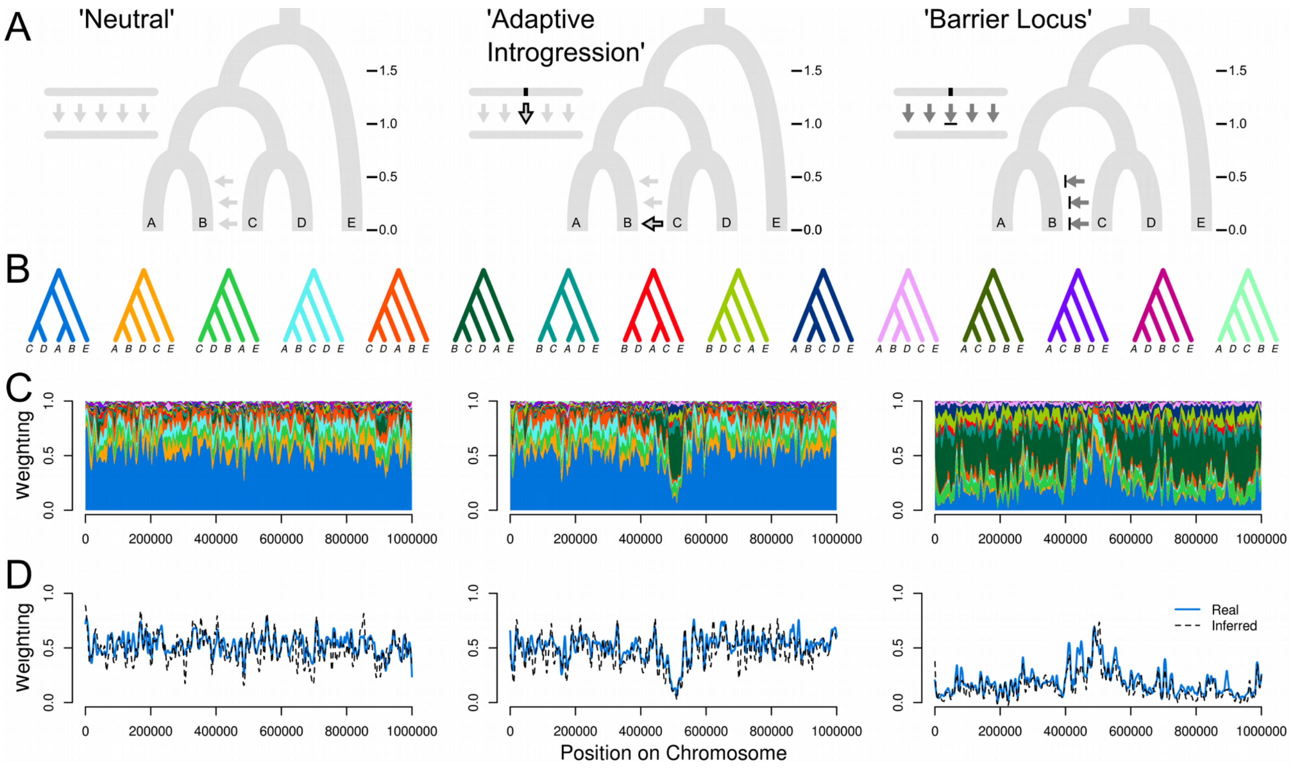

Figure S7. Tests on simulated chromosomes with 5 taxa

(A) In all three demographic scenarios, populations split in the order ((((A,B),C),D),E), at the split times indicated (in units of 4N generations), with migration from C to B. In the 'Neutral' scenario, there is no selection and moderate migration. The 'Adaptive Introgression' scenario is similar, except a beneficial allele at a locus in the centre of the chromosome is allowed to move from population C into B at time 0.1. In the 'Barrier Locus' scenario, the rate of migration is high, but an allele at the central locus that is fixed in C is selected against in population B. **(B)** All fifteen possible taxon topologies. Note that we illustrate the topologies for the four taxa as rooted, with E as the outgroup for simplicity, but the rooting is not considered when computing the weightings. **(C)** Weightings for all topologies plotted (stacked) across the chromosome, with loess smoothing (span = 20 kb). (D) Weightings for topology (((A,B),C),D) inferred from simulated sequence data using non-overlapping 50 SNP windows and neighbour joining. Solid blue lines indicate the true values, and dashed black lines indicate the inferred values. Values are smoothed as above.

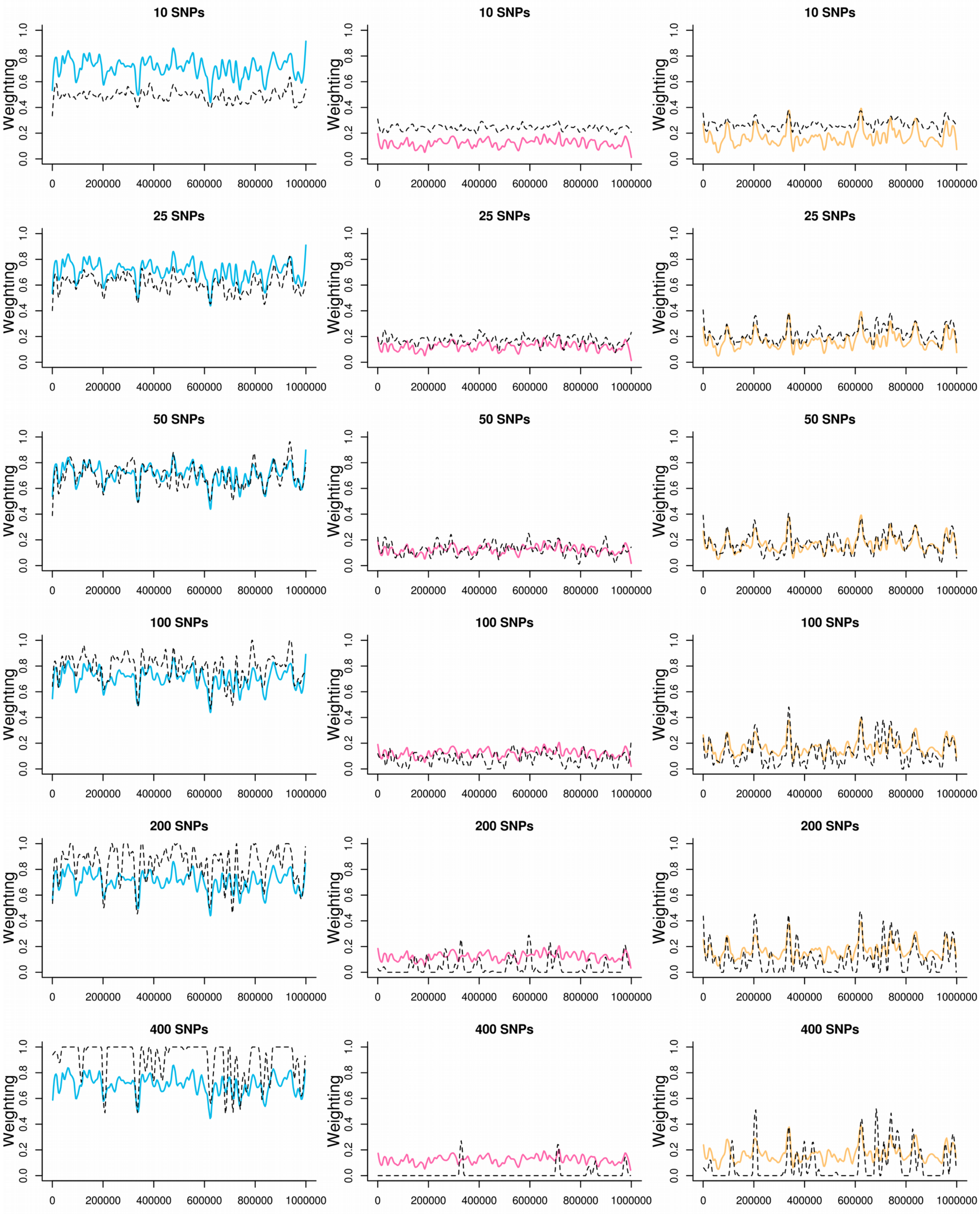

Figure S8. Inferred vs true weightings using different window sizes (ρ = 0.01)

The true (solid coloured line) and inferred (dashed black line) weightings, plotted across the simulated 1 Mb chromosome, with loess smoothing (span 0.04). The three columns with different colours represent the three taxon topologies (see Fig. 2 in the main paper). Rows represent different window sizes (fixed number of SNPs) used for tree inference (neighbour joining).

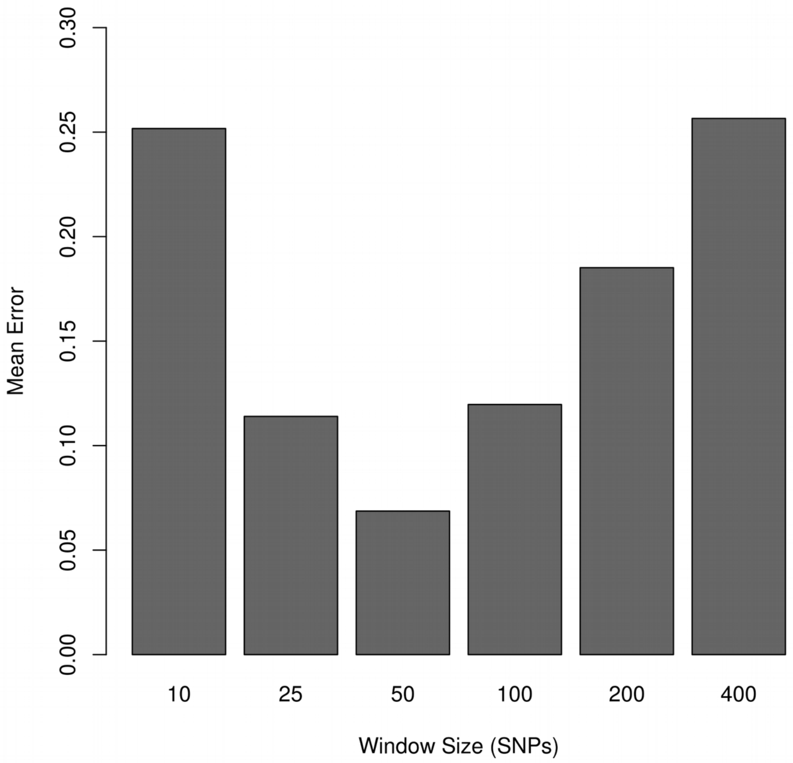

Figure S9. Weighting error rates using different window sizes (ρ = 0.01)

Error rate, calculated as a scaled euclidean distance between the true and observed weightings, averaged over the 1 Mb simulated shromosome. Error rates were computed after first smoothing both the observed and true weightings using loess (span = 0.04).

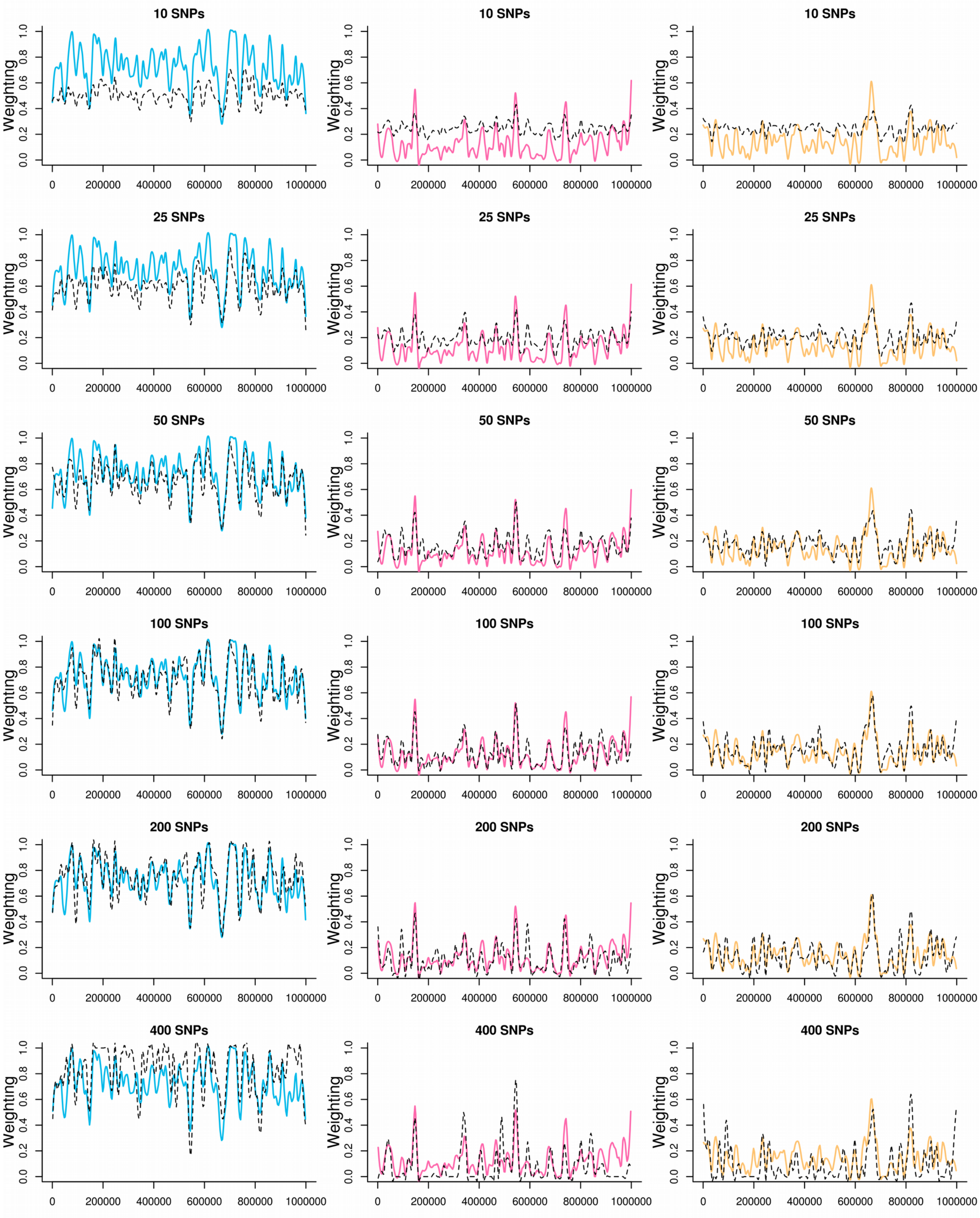

Figure S10. Inferred vs true weightings across simulated chromosomes (ρ = 0.001)

As in Fig. S8, except here simulations used a population recombination rate (ρ) of 0.001.

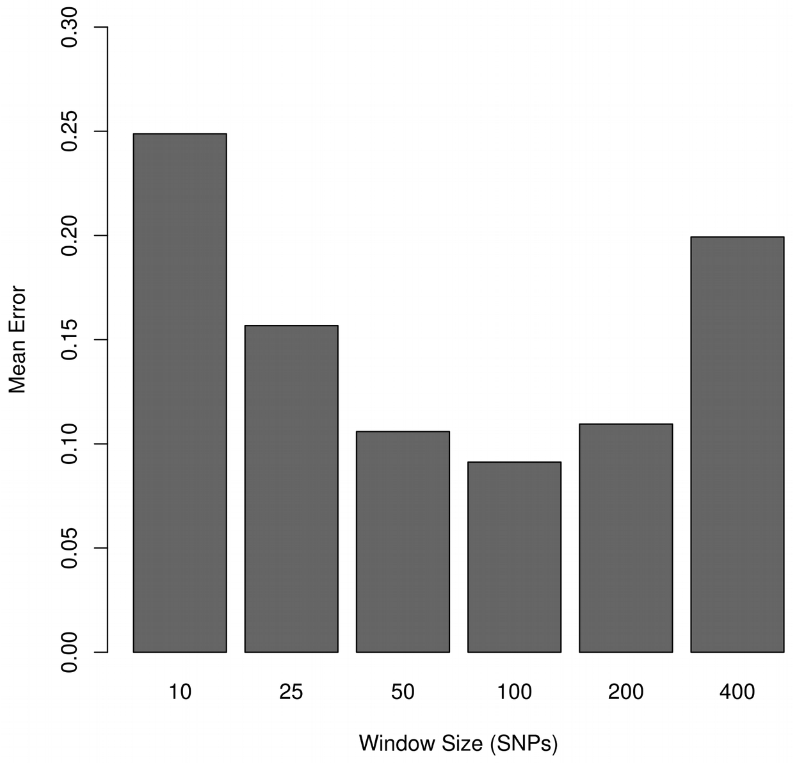

Figure S11. Weighting error rates using different window sizes (ρ = 0.001)

As in Fig. S9, except here simulations used a population recombination rate (ρ) of 0.001.

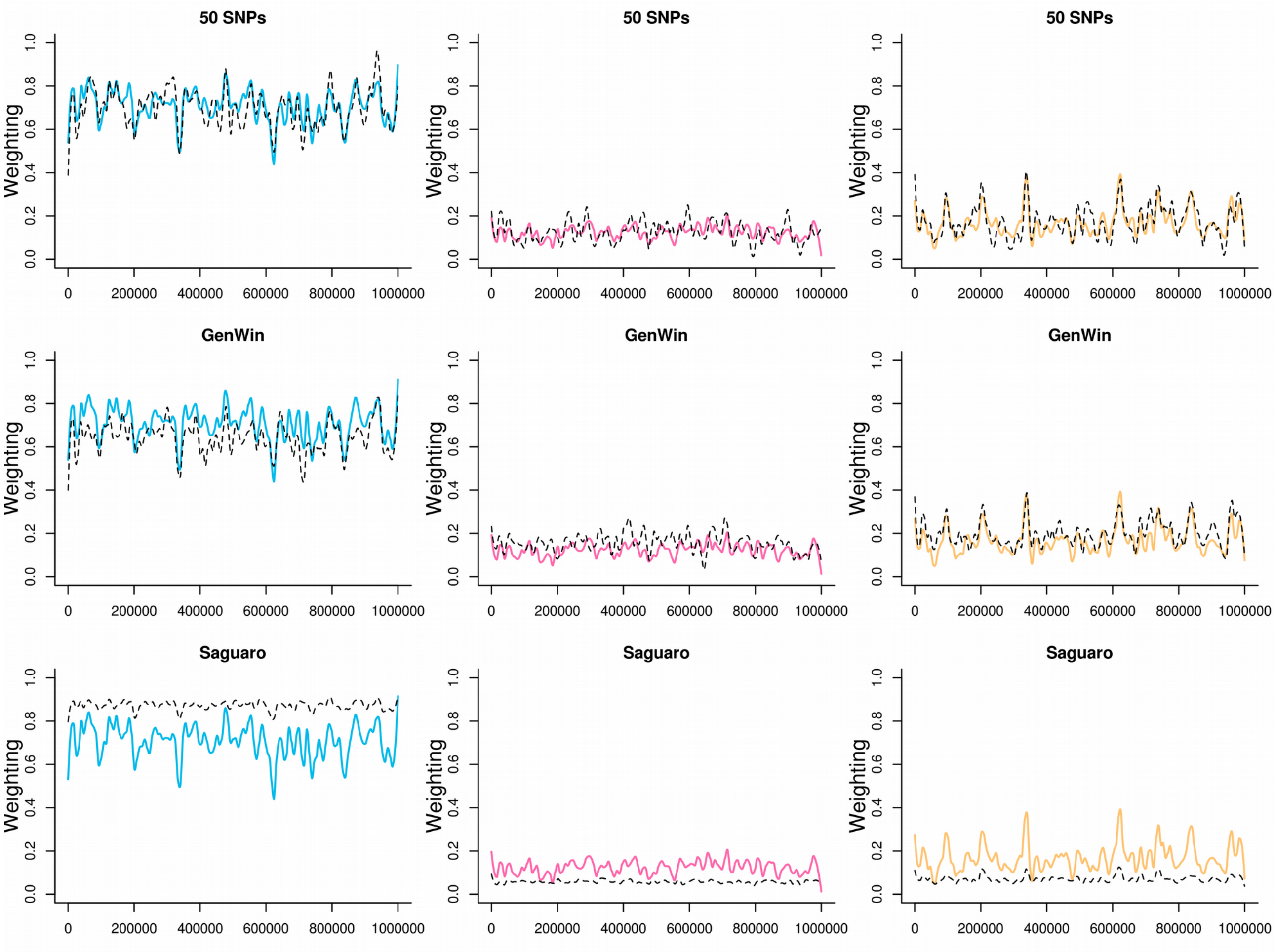

Figure S12. Inferred vs true weightings across simulated chromosomes using different methods for window-based tree inference

As in Fig. S8 and S10, except here comparing different methods for window-based tree inference: 50 SNP windows (first row), *WinGen* inference of breakpoints (second row) or *Saguaro* (row three).

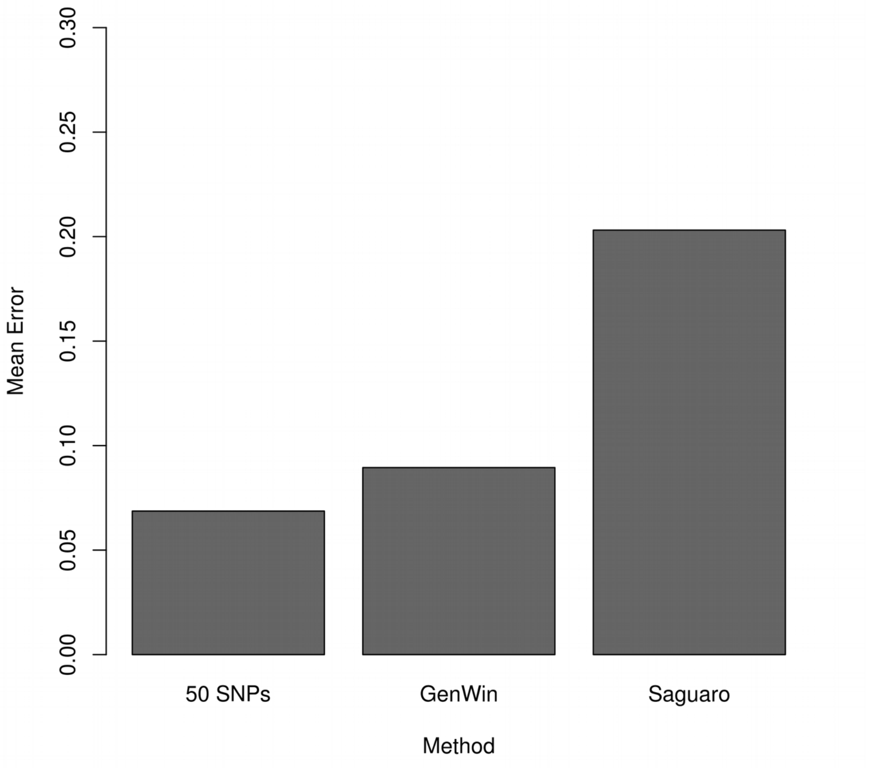

Figure S13. Weighting error rates using different methods for window-based tree inference

As in Fig. S9 and S11, except here comparing different methods for window-based tree inference.

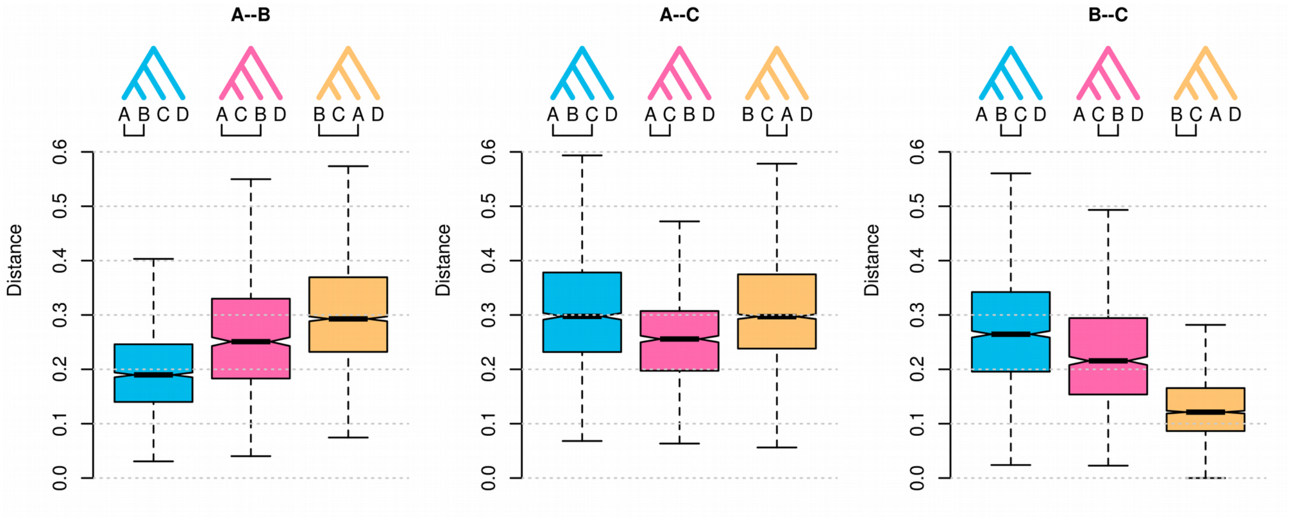

Figure S14. Average branch length separating each pair of taxa in different sub-trees

Boxplots show the distribution of average pairwise distances (i.e. the branch lengths separating each pair of taxa) across all trees, separated by the topology matched by each sub-tree (shown above and colored). For example, the blue box in the left-hand plot gives the distribution of average pair-wise distances between samples from taxon A and B for all sub-trees that matched the topology (((A,B),C),D). The corresponding distances are plotted for the other two topologies in different colors, and then for the other pairwise comparisons (A-C and B-C) in the middle and right-hand plots, respectively. The lower average distance between sequences from B and C (right-hand plot) in topologies where they coalesce first (yellow), compared to the distance between B and A (left-hand plot) in topologies where they coalesce first (blue), suggests that the yellow topology results from recent introgression between B and C, whereas the blue topology matches the population branching order.

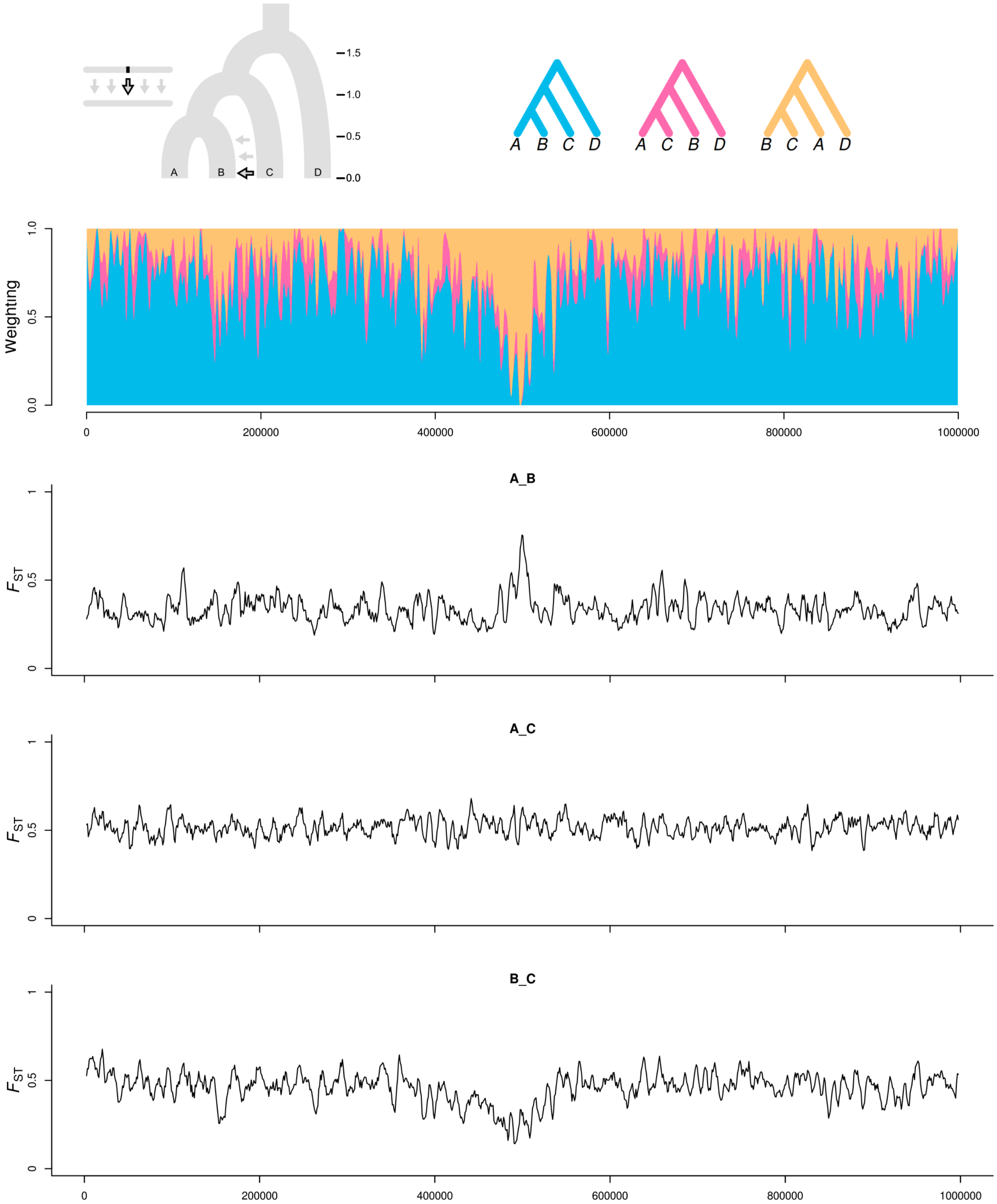

Figure S15. Comparison between topology weighting and F_{ST}

The simulated ‘Adaptive Introgression’ (top) scenario was used to compare topology weighting to F_{ST}. Weightings are plotted as in Fig. 2B except with a loess smoothing (span = 10 kb). Pairwise F_{ST} plots for each pair of ingroup taxa are plotted for 5 kb sliding windows, moving in increments of 1 kb. F_{ST} captures the signal of the adaptive introgression between populations B and C in the form of a peak of divergence between A and B, and marginally reduced divergence between B and C.

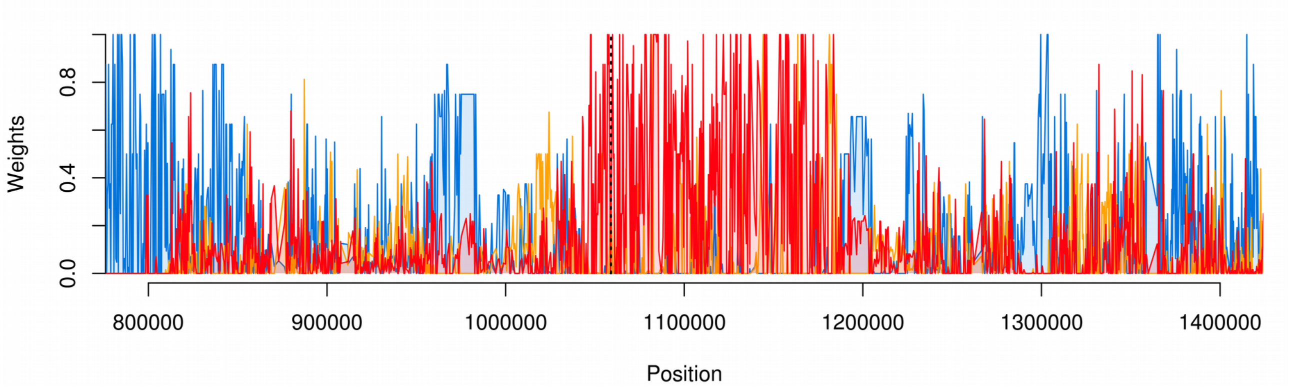

Figure S16. Topology weighting around the *Heliconius optix* gene

Unsmoothed weightings for three topologies from the *Heliconius* analysis, topo3 (blue), topo6 (orange) and topo11 (red) (see Fig. 4 in the main paper for details), are plotted across the region of Chromosome 18 around the gene *optix* (indicated by a dashed black line). A ~150 kb block of high weightings for topo11, which supports introgression between *H. melpomene amaryllis* and *H. timareta thelxinoe*, includes *optix* and its downstream regulatory region that is known to control red wing patterning.
